# Intracerebroventricular delivery of an ODN peptide analog protects dopaminergic neurons and modulates miRNA expression in a mouse model of Parkinson’s disease

**DOI:** 10.3389/fphar.2026.1933852

**Published:** 2026-10-21

**Authors:** Amine Bourzam, Karthi Duraisamy, Omayma Dlimi, Mouna El Mehdi, Amine Cherif, Arnaud Arabo, Gérard Lizard, Taoufik Ghrairi, Benjamin Lefranc, Marie-Anne Le Solliec, Olfa Masmoudi-Kouki, Jérôme Leprince

**Affiliations:** 1 Laboratory of Neuroendocrine, Endocrine and Germinal Differentiation and Communication (NorDiC), Inserm UMR 1239, University of Rouen Normandy, Rouen, France; 2 Laboratory of Neurophysiology, Cellular Physiopathology and Valorization of Biomolecules, LR18ES03, Faculty of Science of Tunis, University of Tunis El Manar, Tunis, Tunisia; 3 Biological Resource Center, Inserm US 51, CNRS UAR 2026, HeRacLeS, University of Rouen Normandy, Rouen, France; 4 Biochemistry of the Peroxisome, Inflammation and Lipid Metabolism (Bio-PeroxIL), EA7270, University of Burgundy, Dijon, France

**Keywords:** Parkinson’s disease, MPTP, ODN, MicroRNAs, neuroprotection, neuroinflammation

## Abstract

**Introduction:**

Parkinson’s disease (PD) is characterized by progressive dopaminergic neuron degeneration, accompanied by oxidative stress, neuroinflammation and apoptosis. Octadecaneuropeptide, ODN, is an astroglia-derived neuropeptide known to exert potent antioxidant and anti‐apoptotic effects, protecting neuronal cells against oxidative damage and inflammation both *in vivo* and *in vitro*. In the present study, we investigated the neuroprotective potential of OP, an iso-active ODN analog, in an acute MPTP‐induced mouse model of PD.

**Methods:**

Mice were assigned to four groups i.e., control, MPTP, OP, and MPTP plus OP. MPTP was administered intraperitoneally (3 × 20 mg/kg, 2‐h intervals), followed 1 h later by an intracerebroventricular injection of the OP (10 ng/10 μL). Brains were collected on day 7 for real-time PCR, Western blotting, immunohistochemistry, and Biochemical analyses.

**Results and Discussion:**

Intracerebroventricular administration of OP significantly prevented the loss of tyrosine hydroxylase expression and mediating neuroprotective effect with stimulation of antiapoptotic gene *Bcl‐2* in the substantia nigra and ventral tegmental area in MPTP‐treated mice. Moreover, OP markedly reduced MPTP‐induced oxidative damage by reducing ROS overproduction and lipid peroxidation and enhancing antioxidant catalase activity. OP also stimulated ERK1/2 phosphorylation and reduced the expression of pro‐inflammatory genes such as *TNF‐α* and increased *IL‐10* mRNA levels. In addition, OP prevented MPTP‐induced down regulation of tight junction proteins *i.e.* occludin, ZO‐1 preserving blood–brain barrier (BBB) integrity. Furthermore, OP abolished MPTP‐induced dysregulation of PD‐related microRNAs including miR‐21‐3p, miR‐29a‐5p and markedly increased miR‐34b‐5p expression in nigrostriatal DA neurons. Taken together, these findings indicate that OP exerts potent neuroprotective effects by reducing oxidative stress, preserving BBB integrity, and limiting neuroinflammation and apoptosis. OP may therefore represent a promising therapeutic candidate for preventing dopaminergic neurodegeneration through modulation of key molecular pathways, including microRNAs involved in oxidative neurodegeneration.

## Graphical Abstract

**Figure:**
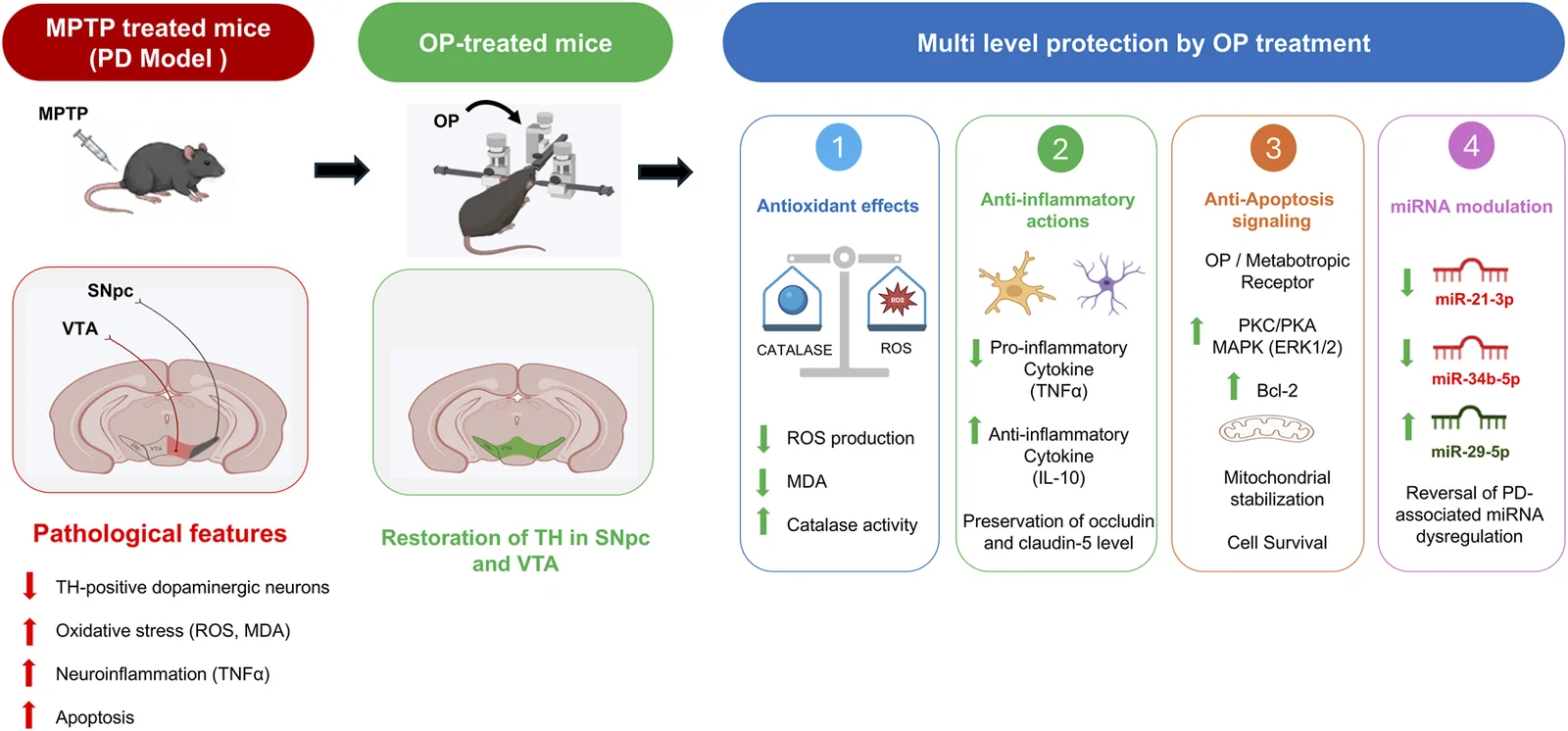
Octadecaneuropeptide analog (OP) protects dopaminergic neurons against MPTP-induced neurodegeneration in a mouse model of Parkinson’s disease. OP restores tyrosine hydroxylase expression in the SNpc and VTA while reducing oxidative stress, neuroinflammation, and apoptosis. Neuroprotection is associated with enhanced catalase activity, ERK1/2 activation, increased Bcl-2 expression. OP also preserves blood‐brain barrier integrity and normalizes PD-related microRNA dysregulation. These findings identify OP as a promising therapeutic candidate for limiting dopaminergic neuron loss through multi-target neuroprotective mechanisms.

## Introduction

Parkinson’s disease (PD) is the second most common neurodegenerative disorder after Alzheimer’s disease, affecting millions of individuals worldwide (Kalia and Lang, 2015; Aarsland et al., 2021; Su et al., 2025). The pathological hallmark of PD is characterized by progressive degeneration of dopaminergic neurons and dopamine depletion in the substantia nigra pars compacta (SNpc), This neuronal loss is accompanied by the formation and accumulation of intraneuronal Lewy bodies, whose major component is misfolded and aggregated α-synuclein, together with other proteins such as parkin and ubiquitin (Braak et al., 1995; Gómez-Benito et al., 2020). This dopaminergic deficit underlies the characteristic motor and non-motor symptoms of PD, including resting tremor, muscular rigidity, akinesia, and bradykinesia (Samii et al., 2004; Tolosa et al., 2021). The etiology of PD is multifactorial and involves a complex interplay between genetic susceptibility and environmental neurotoxin exposure (Gao et al., 2012; Singleton et al., 2013). These factors contribute to oxidative stress, mitochondrial dysfunction, and neuroinflammation, ultimately leading to the progressive loss of dopaminergic neurons (Jenner, 2003; Blesa et al., 2015; Tansey et al., 2022). The increasing prevalence of PD, largely associated with population aging, represents a major public health concern and is accompanied by substantial healthcare and socioeconomic burdens, highlighting the need for effective disease-modifying therapeutic strategies (Li et al., 2025).

Current therapeutic strategies for PD remain largely symptomatic and fail to prevent or slow disease progression. Moreover, their clinical efficacy often declines with long-term treatment, highlighting the need to develop alternative therapeutic approaches with neuroprotective and neurorestorative potential (Francardo et al., 2017; Kim and Lee, 2025). A promising neuroprotective strategy involves the identification and development of neurotrophic factors capable of preserving dopaminergic neuron integrity and function (Francardo et al., 2017; Salamon et al., 2020; Lindholm and Saarma, 2022). Several endogenous neuroprotective peptides, including glial cell line-derived neurotrophic factor (GDNF) and activity-dependent neurotrophic factor (ADNF), have demonstrated strong neuroprotective proprieties in preclinical models and are currently under clinical evaluation for PD (Lindholm and Saarma, 2022; Barker et al., 2020). Accordingly, increasing attention has been directed toward endogenous glial-derived factors, as glial cells play a pivotal role in maintaining neuronal homeostasis and regulating oxidative and inflammatory processes in the central nervous system (CNS) (Lee et al., 2022). Identifying novel astrocyte-derived molecules capable of limiting oxidative stress and modulating pro-inflammatory mediators involved in dopaminergic neurodegeneration therefore represents a key research objective. In this context, the octadecaneuropeptide (ODN), which is exclusively produced by astroglial cells in the CNS of mammals (Tonon et al., 1990; Lanfray et al., 2013; Guillebaud et al., 2020), emerges as a promising candidate due to its potent neuroprotective effects against neurotoxin-induced oxidative stress and apoptosis in neuronal cells (Kaddour et al., 2013; Kaddour et al., 2019; Tonon et al., 2020; Bourzam et al., 2024).

ODN is a neuropeptide produced by astroglial cells and generated through the proteolytic cleavage of an 86-amino acid precursor, the diazepam-binding inhibitor (DBI), also known as acyl-CoA-binding protein (Guidotti et al., 1983). Within the CNS, ODN-like immunoreactivity is widely distributed, with particularly high concentrations observed in brain regions such as the striatum, hippocampus, and hypothalamus (Tonon et al., 2020), which play critical roles in motor control, cognition, and neuroendocrine regulation. Recently, it has been shown that ODN is able to promote neuronal survival and functional recovery in a murin experimental model of ischemic stroke, supporting a key role in neuroprotection and neural tissue repair (Lamtahri et al., 2021). Mechanistically, ODN exerts a potent anti-apoptotic effect through the modulation of key components of the intrinsic apoptotic pathway (Kaddour et al., 2019; Hamdi et al., 2012a; Namsi et al., 2019). In addition, it displays strong antioxidant properties by reducing reactive oxygen species (ROS) overproduction and preventing oxidative stress-induced neuronal damage in both *in vitro* and *in vivo* models (Bahdoudi et al., 2018; Masmoudi-Kouki et al., 2020; Fridhi et al., 2025). Furthermore, studies in DBI knockout mice have shown that the absence of endogenous ODN exacerbates inflammatory responses and oxidative brain damage in the MPTP mouse model of PD, indicating a physiological role for ODN in limiting neuroinflammatory and oxidative processes (Bahdoudi et al., 2018). Collectively, these findings support the view that ODN functions as an astroglia-derived neurotrophic peptide involved in the regulation of neuronal survival and homeostasis under pathological conditions associated with neurodegeneration.

In parallel with therapeutic development, a major objective in PD research is the identification of reliable biomarkers (Mantri et al., 2019). The neuropathological processes underlying PD evolve progressively and often remain clinically silent for years, whereas therapeutic intervention is typically initiated only after the onset of overt motor symptoms, at a stage when substantial dopaminergic neuronal loss has already occurred (Müller, 2025). In this context, non-coding RNAs, particularly microRNAs (miRNAs), have emerged as key molecular regulators implicated in the pathophysiology of PD (O'Brien et al., 2018). MiRNAs modulate post-transcriptional gene expression regulating mitochondrial function, oxidative stress responses, inflammatory signaling and apoptosis (Bartel, 2004; Krol et al., 2010). Accumulating evidence demonstrates that specific miRNAs such as miR-34b, miR-29a and miR-21 are consistently dysregulated in PD patients and experimental models, highlighting their relevance to disease mechanisms (Shaheen et al., 2024). Importantly, these alterations support the potential utility of miRNAs not only as sensitive biomarkers for early disease detection and progression (Shaheen et al., 2024) but also as promising molecular targets for therapeutic strategies aimed at restoring cellular homeostasis and limiting neurodegenerative processes (Han et al., 2025).

In a previous study, we demonstrated that ODN protects cultured astrocytes from oxidative stress-induced injury through modulation of miR-34b, miR-29a, and miR-21 (Bourzam et al., 2024). Noteworthy, the therapeutic application of native ODN *in vivo* is limited by its biochemical instability and unfavorable pharmacokinetic properties, including its inability to cross the blood-brain barrier (BBB) (Garcia de Mateos-Verchere et al., 2001). To overcome these limitations and enable *in vivo* investigation of its neuroprotective potential, we evaluated OP, an isoactive analog designed to retain the biological activity of ODN while exhibiting improved drug-like properties (Leprince et al., 1998; Leprince et al., 2001). Although intracerebroventricular (i.c.v.) administration was used to ensure central delivery, OP represents a more suitable candidate for *in vivo* studies as previously reported (Guillebaud et al., 2020; Fridhi et al., 2025). In the present study, we hypothesized that OP exerts robust neuroprotective effects against MPTP-induced dopaminergic neurodegeneration through the coordinated regulation of oxidative stress, neuroinflammatory responses, apoptotic signaling pathways, and miRNA expression. We provide the first *in vivo* evidence that OP preserves dopaminergic neurons in the SNpc, attenuates oxidative stress and neuroinflammation, and restores molecular signatures associated with neuronal survival and miRNA-mediated regulatory mechanisms.

## Materials and methods

### Animals

Ten-week-old C57BLJ/6 male mice were purchased from Charles Rivers Laboratories (L’Obrestle, France) and housed, 3–5 per cage, at the Biological Resources Service of the HeRacLeS research infrastructure, (Inserm US 51, CNRS UAR 2026; Rouen, France). Animals were maintained in a ventilated, temperature-controlled room (22 °C ± 1 °C) under a 12 h light/dark photoperiod cycle (lights on between 07:00 a.m. and 07:00 p.m.) and with free access to food and water. All experiments were performed between 07:30 a.m. and 06:00 p.m. in testing rooms adjacent to the animal facility, using naïve cohorts of mice that were habituated to the experimental procedures prior to the beginning of experiments. All experimental procedures were approved by the Normandy Regional Ethics Committee (Agreement of the Ministry of Research n°54, APAFIS n°: 2021020814212189) and conducted by authorized investigators in accordance with Council Directive 86/609/EEC of 24 November 1986.

### *In vivo* treatment and experimental design

Animals were randomly assigned to four experimental groups: Control (n = 8), MPTP (n = 8), OP (n = 8), and MPTP + OP (n = 8). The OP full sequence (RPGLLDLK) is a synthetic analog of ODN (Leprince et al., 1998; Leprince et al., 2001). On the first day of treatment (D0), mice in the MPTP-treated groups received three intraperitoneal (i.p.) injections of MPTP (20 mg/kg in 100 μL of 0.9% NaCl) at 2-h intervals. In addition, mice in the OP-treated groups received a single i.c.v. injection of OP (10 ng in 10 µL of 0.9% NaCl) administered 1 h after the last MPTP injection (Figure 1). Control animals received equivalent volumes of saline (0.9% NaCl) instead of MPTP and/or OP, according to their assigned group. 7 days after the beginning of the treatment (D7), mice were deeply anesthetized and euthanized. Brains were collected for subsequent real-time qPCR, Western blot, immunohistochemistry, and biochemical analyses.

**FIGURE 1:**
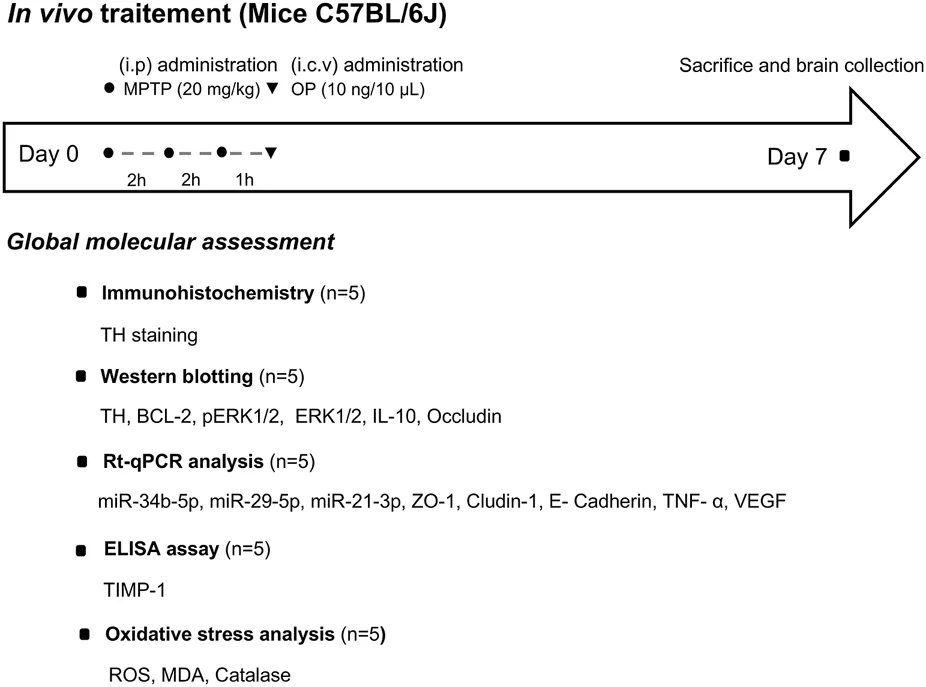
Experimental design.

### Intracerebroventricular injections

Adult mice were anesthetized by i. p. injection of a ketamine/xylazine mixture (100/10 mg/kg, 100 μL), administered in the lower right or left abdominal quadrant of the conscious, restrained animal. Adequate depth of anesthesia was confirmed by the absence of paw withdrawal reflex in response to a toe pinch. Mice were then secured in a stereotaxic frame, and the scalp was shaved and disinfected with betadine solution before being incised (∼1.5 cm). The skull was exposed and cleaned, and a craniotomy was performed using a stereotaxic drill at coordinates corresponding to a lateral ventricle. A sterile 30G needle was lowered into the lateral ventricle, and 10 μL of either sterile saline (0.9% NaCl) or OP (10 ng) was injected at a rate of 10 μL/min. Following i.c.v. injection, the needle was slowly withdrawn, and the incision was closed with 5–7 sutures with 5.0 silk thread. The surgical site was disinfected again with betadine and covered with 5% lidocaine cream. Mice received a single subcutaneous perioperative injection of ketoprofen (5 mg/kg, 0.1 mL) for analgesia prevention. This procedure lasted approximately 25–30 min. Animals were then returned to individual cages equipped with environmental enrichment and closely monitored during recovery, particularly over the first hour post-surgery. Post-operative welfare was assessed regularly to ensure proper recovery and to detect any signs of discomfort.

### Immunohistochemistry

Animals were anesthetized with isoflurane and intracardially perfused with 0.9% NaCl to clear the vascular system, followed by 4% paraformaldehyde (PFA; Sigma-Aldrich, Saint-Quentin-Fallavier, France) for tissue fixation. Brains were then removed and post-fixed overnight in a 4% PFA solution at 4 °C. Coronal brain sections (50 μm thick) were obtained at the levels of the SNpc using a vibratome (Leica VT1000 S, Leica Biosystems, Nussloch, Germany). Free-floating sections were collected in 6-well plates containing PBS supplemented with 0.02% sodium azide and stored at 4 °C. For immunohistochemistry, three to four sections per brain were preincubated for 1 h at room temperature in a blocking solution containing normal donkey serum (1:50), 1% bovine serum albumin, and 0.3% Triton X-100 (VWR International, Strasbourg, France) in PBS. Sections were then incubated overnight at 4 °C with a rabbit anti-tyrosine hydroxylase (TH) primary antibody (1:1000, Merck Millipore, Billerica, MA, United States). After three washes in PBS, sections were incubated for 90 min at room temperature with Alexa 488- or Alexa 594-conjugated donkey anti-rabbit IgG secondary antibodies (1:1000, Invitrogen Life Technologies, Longjumeau, France). Finally, sections were rinsed three times in PBS, mounted onto slides, and cover-slipped with Eukkit mounting medium (Merck Millipore). Fluorescence images were acquired using a Leica THUNDER Imager (computational clearing; LAS X software, Wetzlar, Germany). Quantitative analysis of fluorescence intensity was performed using ImageJ software.

### Total RNA extraction and quantitative RT-qPCR

Total RNA was extracted from the SNpc of mice 7 days after MPTP treatment using a NucleoSpin RNA kit (Macherey-Nagel, Hoerd, France), according to the manufacturer’s instructions. RNA concentration and purity were assessed prior to reverse transcription. cDNA was synthetized from 1 μg of total RNA using the SensiFAST cDNA kit (Meridian Bioscience, London, United Kingdom). Quantitative RT-PCR was performed using an ABI PRISM 7500 Sequence Detection System (Applied Biosystems, Courtaboeuf, France) with One Green FAST qPCR Premix (OZYME, Saint-Cyr-l’École, France) and gene-specific forward and reverse primers (Table 1). Relative gene expression levels were calculated using the comparative cycle threshold (Ct) method (2^−ΔΔCT^), with GAPDH used as the internal reference gene.

**TABLE 1:** Sequences of the primers used for real-time qPCR experiments.

Gene	GenBank accession number	Sequence
*ZO-1*	NM_009386.3	​
	Forward	5-ACC​CGA​AAC​TGA​TGC​TGT​GGA​TAG-3
	Reverse	5-AAA​TGG​CCG​GGC​AGA​ACT​TGT​GTA-3
*Claudin-1*	NM_016674.4	​
	Forward	5-AGC​TGC​CTG​TTC​CAT​GTA​CT-3
	Reverse	5- CTC​CCA​TTT​GTC​TGC​TGC​TC-3
*E-cadherin*	NM_001317184.2	​
	Forward	5-GGT​TTT​CTA​CAG​CAT​CAC​CG-3
	Reverse	5-GCT​TCC​CCA​TTT​GAT​GAC​AC-3
*TNF-a*	NM_013693.3	​
	Forward	5-TAC​TGA​ACT​TCG​GGG​TGA​TTG​GTC​C-3
	Reverse	5- CAG​CCT​TGT​CCC​TTG​AAG​AGA​ACC-3
*VEGF*	NM_001025250.3	​
	Forward	5-GCG​GAT​CAA​ACC​TCA​CCA​AA-3
	Reverse	5-TTC​ACA​TCG​GCT​GTG​CTG​TAG-3
*GAPDH*	NM_001289726.1	​
	Forward	5-ACT​TTG​TCA​AGC​TCA​TTC​C-3
	Reverse	5-TGC​AGC​GAA​CTT​TAT​TGA​TG-3

miRNAs were extracted and purified using the mirVana™ miRNA isolation kit (Thermo Fisher, Waltham, MA, United States), according to the manufacturer’s instructions. RNA quantity and quality were assessed using a Nanodrop One Spectrophotometer and a Qubit 4 Fluorometer. Reverse transcription and qPCR were performed using the TaqMan microRNA reverse transcription kit and TaqMan fast advanced master mix (Thermo Fisher Scientific). Specific TaqMan probes were used for miR-34b-5p, miR-29a-5p, and miR-21-3p (002617, 002447, and 002493, respectively). U6 snRNA (001973, Thermo Fisher) was used as an internal control for normalization. Quantitative RT-PCR was performed on QuantStudio 5. Relative expression levels were calculated using the Ct method as stated above.

### Western blot analysis

After euthanasia, the SNpc and ventral tegmental area (VTA) were rapidly dissected from each animal. Tissue samples were homogenized in N-PER neuronal protein extraction reagent supplemented with phosphatase I, II, and III inhibitor cocktails (Thermo Fisher), according to the manufacturer’s instructions. Homogenates were centrifuged at 10,000 × *g* for 10 min at 4 °C, and the supernatant containing soluble proteins were collected. Protein concentrations were determined using the Bradford assay and normalized across samples. Proteins were finally denatured at 95 °C for 5 min in sample buffer containing 50 mM Tris-HCl (pH 7.5), 20% glycerol, 0.7 M 2-mercaptoethanol, 0.004% (*w*/*v*) bromophenol blue, and 3% (*v*/*v*) SDS.

Equal amounts of protein (∼25 μg) were separated by 12% SDS/polyacrylamide gel electrophoresis (SDS-PAGE) and transferred onto nitrocellulose membranes (Bio-Rad Laboratories, Hercules, CA, United States). Membranes were first blocked for 1 h at room temperature in a solution containing 5% skim milk in 50 mM Tris-buffered saline supplemented with 0.1% Tween 20 (TBST), and then incubated overnight at 4 °C with primary antibodies against target proteins (Table 2). Following three washes in TBST, membranes were incubated for 1 h at room temperature with an HRP-conjugated secondary antibody (anti-rabbit for target proteins and anti-mouse for GAPDH) diluted in TBST containing 5% skim milk. Membranes were washed again, and immunoreactive bands were revealed using the Calrity Western ECL substrate kit (Bio-Rad Laboratories). Signal acquisition and quantification were performed using Image Lab 6.0.1.

**TABLE 2:** Antibodies used for Western blotting experiments.

Antibody	Dilution	MW (kDa)	References	Supplier/Company
TH	1:1,000	62	AB152	Merck Millipore
Bcl-2	1:1,000	26	AF6139	Affinity Biotech
ERK1/2	1:1,000	44/42	4,370	Cell signaling Technology
pERK1/2	1:1,000	44/42	4,695	Cell signaling Technology
IL-10	1:1,000	26/22	Ab9969	Abcam
Occludin	1:1,000	59	224,526	Abcam
GAPDH	1:50,000	37	A19056	ABclonal
Anti-rabbit IgG-HRP	1:5,000	—	Sc-2004	Santa Cruz Biotechnology
Anti-mouse IgG-HRP	1:5,000	—	Sc-2005	Santa Cruz Biotechnology

For sequential probing of multiple proteins on the same membrane, a stripping step was performed according the manufacturer’s protocol. Membranes were incubated in Restore Western blot stripping buffer (Bio-Rad) for 10 min at room temperature with gentle agitation, followed by two washes in PBS and two washes in TBST (5 min each). Membranes were then re-blocked in 5% skim milk in TBST for 1 h at room temperature, and re-incubated with the next primary antibody using the same procedure described above.

### Quantification of TIMP-1 levels by ELISA

Mouse plasma samples (100 μL) were analyzed for TIMP-1 levels using a commercially available ELISA kit (ab196265, Abcam). Quantification was performed in accordance with the manufacturer’s instructions. Absorbance was measured at 450 nm using a FlexStation 3 microplate reader (Molecular Devices, Sunnyvale, CA, United States).

### Assessment of oxidative stress and antioxidant enzyme activities

ROS production was assessed by measuring the fluorescence of 2′,7′-dichlorofluorescein (DCF), generated from the deacetylation and subsequent oxidation of the non-fluorescent compound DCFH-DA. Briefly, 100 μL of brain tissue extract from SNpc were incubated with 10 μM DCFH-DA for 30 min at 37 °C in the dark. Fluorescence was measured using a FL800TBI fluorescence microplate reader (Bio-Tek Instruments, Winooski, VT, United States) with excitation and emission wavelengths set at 485 and 538 nm, respectively.

Lipid peroxidation was evaluated by quantifying malondialdehyde (MDA) levels using the spectrophotometric method (Ohkawa et al., 1979). Briefly, 20 µL of brain homogenate (∼50 µg total protein) were mixed with 125 µL of 20% trichloroacetic acid and 50 µL of 0.67% thiobarbituric acid (TBA). Samples were incubated for 30 min at 95 °C and then rapidly cooled on ice. Subsequently, 800 µL of butanol were added, followed by vortexing and centrifugation at 3,000 × g for 10 min at 4 °C. The organic phase containing the MDA-TBA_2_ adduct was collected, and absorbance was measured at 532 nm using a UV-visible spectrophotometer. MDA concentrations were calculated using the molar extinction coefficient ε = 1.65 × 10^5^ M^−1^·cm^−1^ and expressed as nmol MDA per mg of total protein, as determined by the Bradford assay.

Catalase activity was determined using a spectrophotometric method (Aebi, 1974), based on the decomposition of H_2_O_2_ monitored at 240 nm. The reaction mixture (1 mL total volume) contained 980 µL of 50 mM phosphate buffer (pH 7.0), 10 µL of brain extract, and 10 µL of H_2_O_2_ (final concentration: 30 mM). The decrease in H_2_O_2_ absorbance at 240 nm was recorded for 3 min at 30 s intervals using a UV-visible spectrophotometer equipped with 1 cm quartz cuvettes at 25 °C. Catalase activity was calculated from the initial rate of absorbance change (ΔA_240_/min) using the molar extinction coefficient of H_2_O_2_ (ε = 40 M^−1^·cm^−1^) and expressed as nmol H_2_O_2_ decomposed per min per mg of protein, normalized to the control group.

### Statistical analysis

Data are expressed as mean ± standard error of the mean (SEM). Statistical analyses were performed by using GraphPad PRISM 10.5.0 software (GraphPad Software, San Diego, CA, United States). Differences between groups were evaluated by one-way ANOVA followed by Bonferroni’s *post hoc* test. A *p* value ≤ 0.05 was considered statistically significant.

## Results

### OP prevents MPTP-induced loss of dopaminergic neurons by restoring TH expression in the SNpc

MPTP administration induced a decrease in body weight (−20%, *p* < 0.05) within 2 days of treatment initiation, with values tending to return toward control levels by D7. A single i.c.v. administration of OP (10 ng, 10 µL), which did not significantly affect body weight relative to control group, prevented MPTP-induced weight loss, resulting in a weight trajectory comparable to that observed in control animals (Figure 2). Although body weight in MPTP + OP-treated group remained non-significantly lower than in the control and OP-treated groups, it was significantly higher than in the MPTP group at D4 (Figure 2).

**FIGURE 2:**
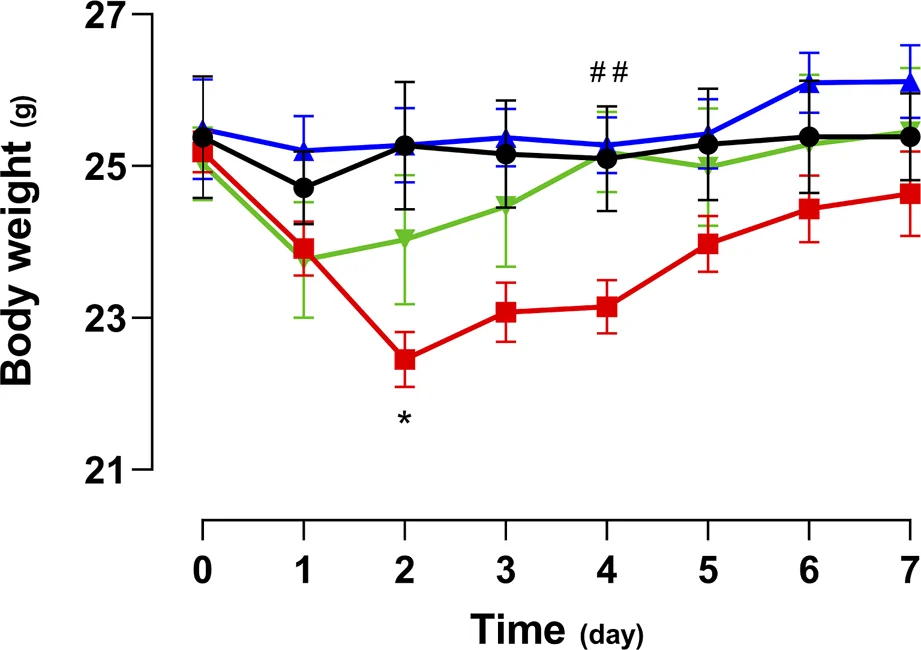
Time-course of OP effect on body weight in MPTP-treated mice. On D0, mice received 3 i. p. injections of saline (0.9% NaCl; control, black circles; OP, blue triangles) or MPTP (20 mg/kg; MPTP, red squares; MPTP + OP, green inverted triangles) at 2 h of interval. One hour after the final i. p. injection, animals were given an i.c.v. injection of saline (control, black circles; MPTP, red squares) or OP (10 ng, 10 μL; OP, blue triangles; MPTP + OP, green inverted triangles). Body weight of each mouse was recorded daily from D0 to D7. Data are expressed as mean ± SEM from eight animals per group. Statistical analysis was performed using one-way ANOVA followed by Bonferroni’s *post hoc* test. *p < 0.05 vs. saline-treated mice; ##*p* < 0.01 vs. MPTP-treated mice.

To assess the neuroprotective capacity of OP against MPTP-induced dopaminergic neuronal loss, immunohistochemical and Western blot analyses were conducted (Figure 3). Consistent with previous studies (Bahdoudi et al., 2018; Fridhi et al., 2025; Meredith and Rademacher, 2011), acute MPTP administration (20 mg/kg, three injections at 2-h intervals) resulted in a significant reduction in TH-immunoreactive neurons both on SNpc and VTA compared to saline-treated animals (Figures 3A-a,b,B), indicating a loss of dopaminergic neurons within 7 days post-treatment. Quantitative analysis revealed a 1.61-folddecrease in mean TH fluorescence intensity in MPTP-treated animals compared to sham animals (Figure 3B). Western blot analysis further confirmed a significant decrease in TH protein levels in the SNpc (1.86-fold, Figure 3C) and VTA (1.3-fold, Figure 3D) of MPTP-treated mice. Single i.c.v. administration of OP (10 ng, 10 μL) did not significantly affect TH levels on its own, but significantly attenuated MPTP-induced dopaminergic neuronal loss in both the SNpc and VTA (Figures 3A-c,d,B–D). Quantification of TH fluorescence mean intensity in the SNpc showed that OP counteracted the MPTP-induced reduction in TH-immunoreactive neurons by 1.19-fold (Figure 3B). Western blot analysis further demonstrated that OP treatment significantly prevented the MPTP-evoked decrease in TH protein levels in the SNpc (1.24-fold vs. MPTP group) and VTA (1.41-fold vs. MPTP group) (Figures 3C,D), demonstrating that OP protected dopaminergic neurons from degeneration in our MPTP-induced mouse model of PD.

**FIGURE 3:**
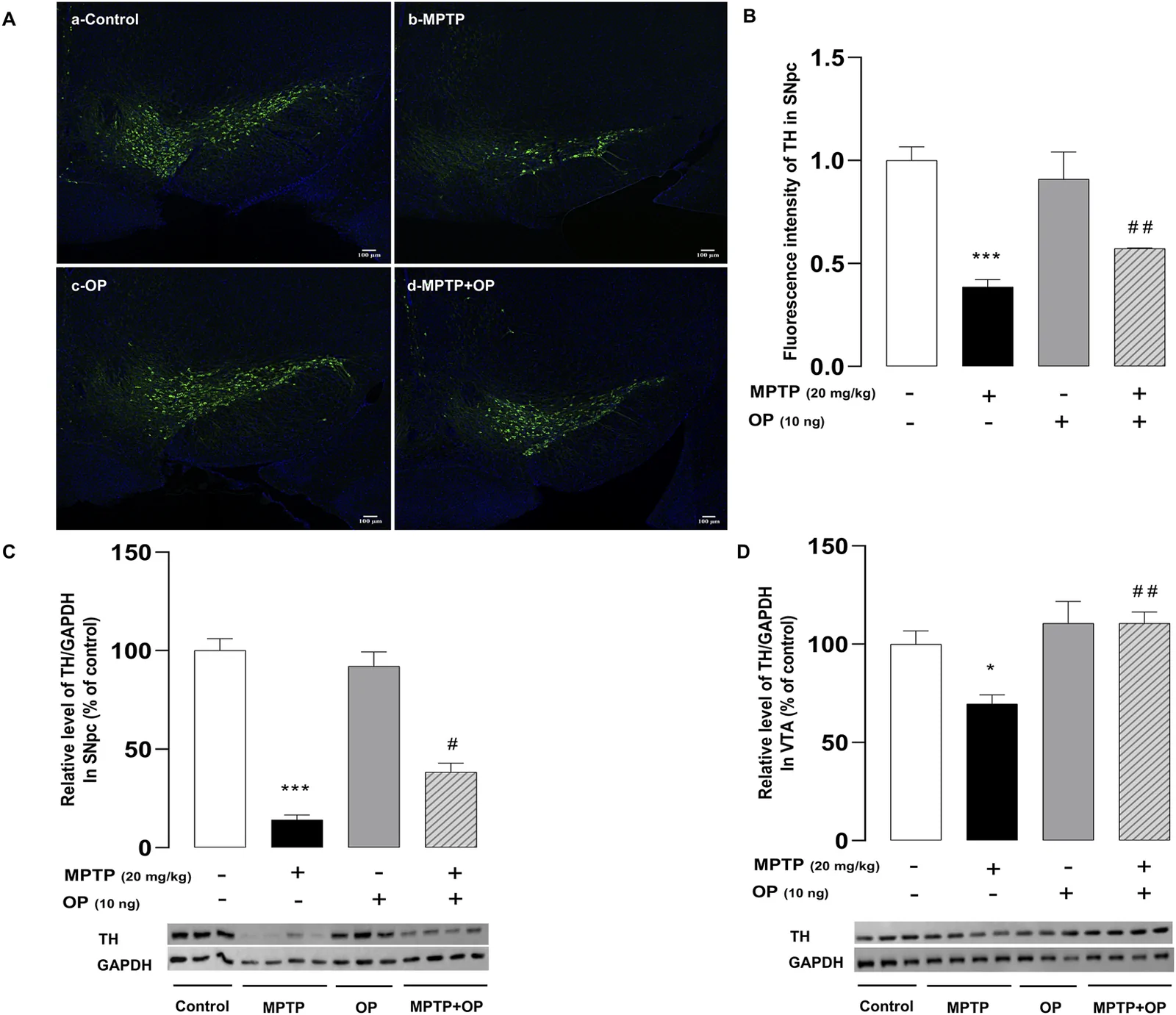
OP reverses TH expression in MPTP-treated mice. **(A)** Representative images of TH immunostaining in the midbrain 7 days after treatment with saline (0.9% NaCl; control; **(A)**, MPTP (3 mg/kg × 20 mg/kg; **(B)**, OP (10 ng, 10 μL; **(C)** and MPTP + OP **(D)**. Scale bar = 100 μm. SNpc, substantia nigra pars compacta; SNpr, substantia nigra pars reticulata; VTA, ventral tegmental area. **(B)** Quantification of TH immunofluorescence intensity in the SNpc 7 days after MPTP treatment in the presence or absence of OP. **(C,D)** Densitometric analysis of TH protein levels in the SNpc and VTA of control, MPTP, OP, and MPTP + OP-treated mice. Representative immunoblots and corresponding quantifications normalized to GAPDH are shown. Data are expressed as mean ± SEM from three animals for immunohistochemistry and five animals for Western blot. Statistical analysis was performed by one-way ANOVA followed by Bonferroni’s *post hoc* test. *p < 0.05, ***p < 0.001 vs. saline-treated mice; #*p* < 0.05, ##*p* < 0.01 vs. MPTP-treated mice.

### OP prevents MPTP-induced ROS accumulation and oxidative damage

Since MPTP induces oxidative stress that contributes to neurodegeneration, we examined the effects of OP on ROS production and MDA levels in SNpc homogenates from MPTP-treated mice. Using a ROS-sensitive fluorescent probe, we found that MPTP-treated animals exhibited a significant increase in ROS levels in the SNpc (1.51-fold) compared to saline-treated mice (Figure 4A). This increase in ROS was accompanied by a marked elevation of MDA levels (3.69-fold), a product of polyunsaturated fatty acid oxidation (Figure 4B). Co-administration of OP with MPTP markedly reduced ROS overproduction (0.28-fold; Figure 4A) and MDA levels (0.4-fold relative to MPTP; Figure 4B). Moreover, OP administration significantly increased catalase activity in the SNpc of MPTP-exposed mice (1.75-fold; Figure 4C), indicating a stimulation of endogenous antioxidant defense that may contribute to protection against MPTP-induced oxidative damage.

**FIGURE 4:**
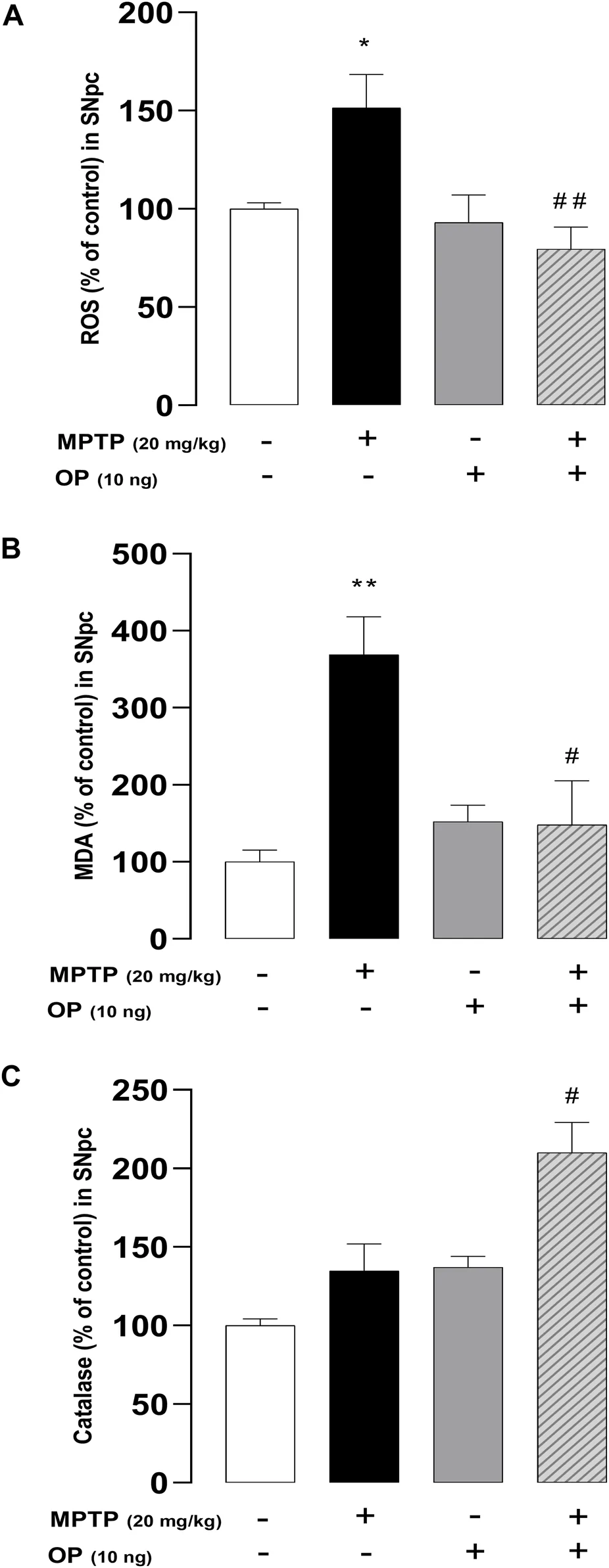
OP attenuates oxidative stress in MPTP-treated mice. Quantification of **(A)** ROS accumulation, **(B)** MDA accumulation, and **(C)** catalase activity in SNpc 7 days after treatment with saline (0.9% NaCl), MPTP (3 mg/kg × 20 mg/kg), OP (10 ng, 10 μL) and MPTP + OP. Data are expressed as mean ± SEM from five animals. Statistical analysis was performed by one-way ANOVA followed by Bonferroni’s *post hoc* test. *p < 0.05, **p < 0.01 vs. saline-treated mice; #*p* < 0.05; ##*p* < 0.01 vs. MPTP-treated mice.

### OP protects against MPTP-induced apoptosis through Bcl-2 and ERK activation

To investigate the ability of OP to prevent MPTP-induced cell death, the levels of key anti-apoptotic proteins were examined in the SNpc and VTA. Western blot analysis revealed a non-significative reduction in Bcl-2 protein levels in both the SNpc (0.74-fold) and the VTA (0.72-fold) following MPTP treatment (Figures 5A,B). Noteworthy, OP i.c.v. administration alone (10 ng, 10 µL) did not significantly alter Bcl-2 protein levels in either the SNpc or VTA compared with control animals. In contrast, single i.c.v. administration of OP in toxin-treated mice significantly increased Bcl-2 protein levels in the SNpc (1.48-fold) and VTA (1.54-fold), these findings suggest that modulation of anti-apoptotic signaling may contribute to the neuroprotective effects of OP To further elucidate the molecular mechanisms underlying the neuroprotective action of OP, we next examined its effects on the activation of MAPK/ERK signaling pathway. OP administration alone significantly increased total ERK1/2 protein levels in the SNpc (1.68-fold, Figure 5C) and VTA (+48%, Figure 5D), whereas MPTP treatment had no effect. This increase was accompanied by a parallel enhancement of ERK1/2 phosphorylation in both regions (1.68-fold in the SNpc and 1.48-fold in the VTA; Figures 5E,F), indicating activation of MAPK/ERK signaling. MPTP-treated animals exhibited a marked reduction in ERK1/2 phosphorylation in the VTA (0.5-fold vs. control), whereas no significant change was observed in the SNpc (Figures 5E,F). In the presence of MPTP, OP maintained its stimulatory effect on ERK1/2 signaling, preserving elevated levels of both ERK1/2 and phosphorylated ERK1/2 in both the SNpc and VTA (1.65-fold and 2.66-fold, respectively; Figures 5E,F).

**FIGURE 5:**
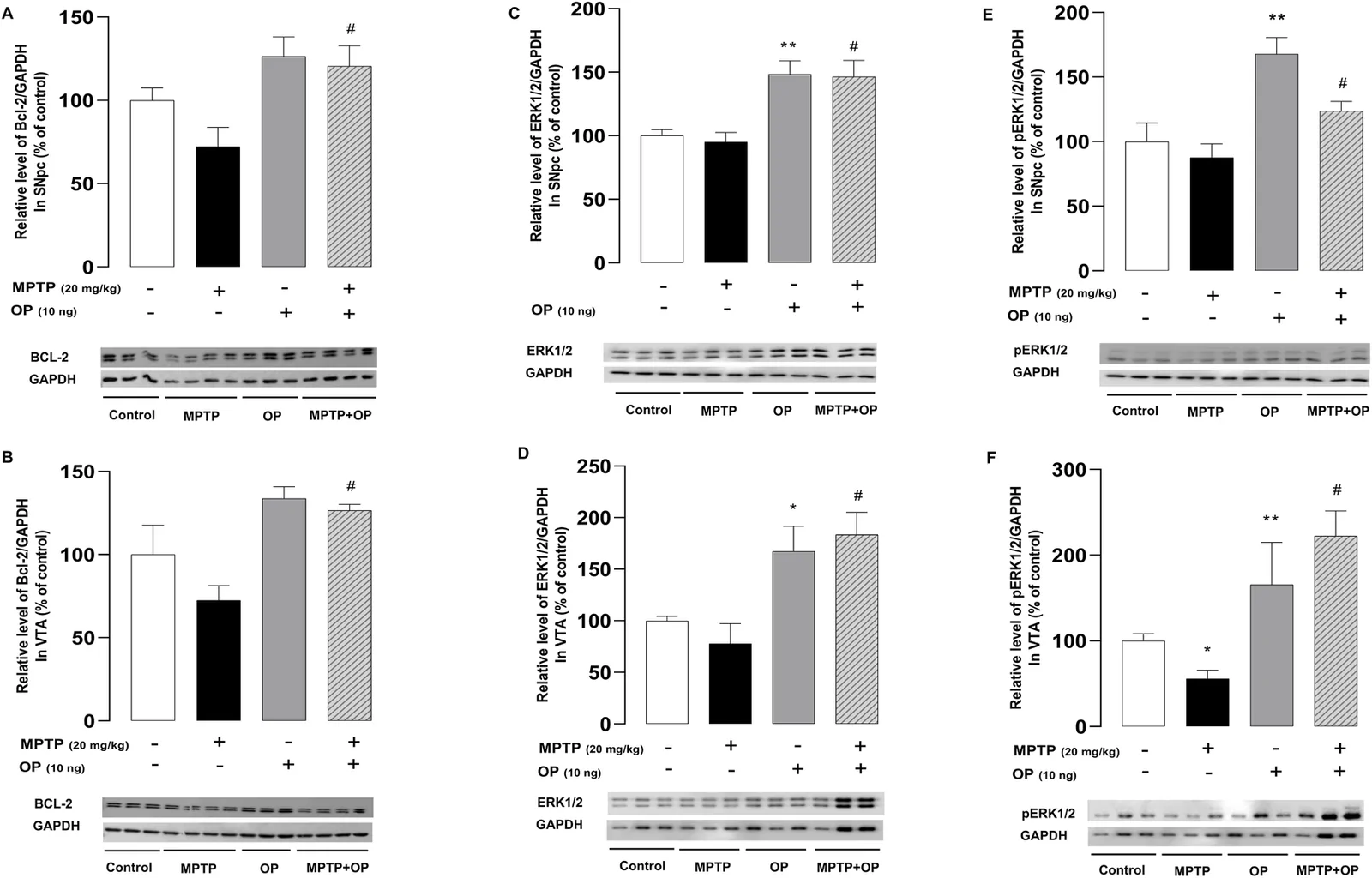
OP modulates apoptotic and MAPK signaling pathways in MPTP-treated mice. Densitometric analysis of Bcl-2, ERK1/2, and pERK1/2 protein levels in the SNpc **(A,C,E)** and VTA **(B,D,F)** of control, MPTP, OP, and MPTP + OP-treated mice. Representative immunoblots and corresponding quantifications normalized to GAPDH are shown. Data are expressed as mean ± SEM from five animals. Statistical analysis was performed by one-way ANOVA followed by Bonferroni’s *post hoc* test. *p < 0.05, **p < 0.01 vs. saline-treated mice; #*p* < 0.05 vs. MPTP-treated mice.

### OP modulates IL-10 and maintains occludin levels in MPTP-induced neuroinflammatory conditions

Given that MPTP induces a robust neuroinflammatory response contributing to dopaminergic neurodegeneration, we investigated whether OP modulates anti-inflammatory and BBB related markers in MPTP-treated mice. In the SNpc, Western blot analysis showed that IL-10 protein levels were not significantly altered 7 days after MPTP administration (Figure 6A). In contrast, single i.c.v. administration of OP in MPTP-treated mice and alone significantly increased IL-10 protein levels (2.43-fold vs. control; 2.99-fold vs. MPTP; Figure 6A). In the VTA, MPTP treatment did not significantly modify IL-10 expression compared to the control groups (Figure 6B). Even for the co-treatment with MPTP and OP did not alter IL-10 levels compared to MPTP alone, indicating no additional effect of the combined treatment (Figure 6B). Regarding BBB-associated proteins, OP markedly enhanced occludin levels in the SNpc (1.88-fold vs. control) and maintained them above those observed in MPTP-treated mice (+92% vs. MPTP; Figure 6C), suggesting preservation of BBB integrity. In the VTA, occludin levels were not significantly affected by either MPTP and/or OP treatment (Figure 6D).

**FIGURE 6:**
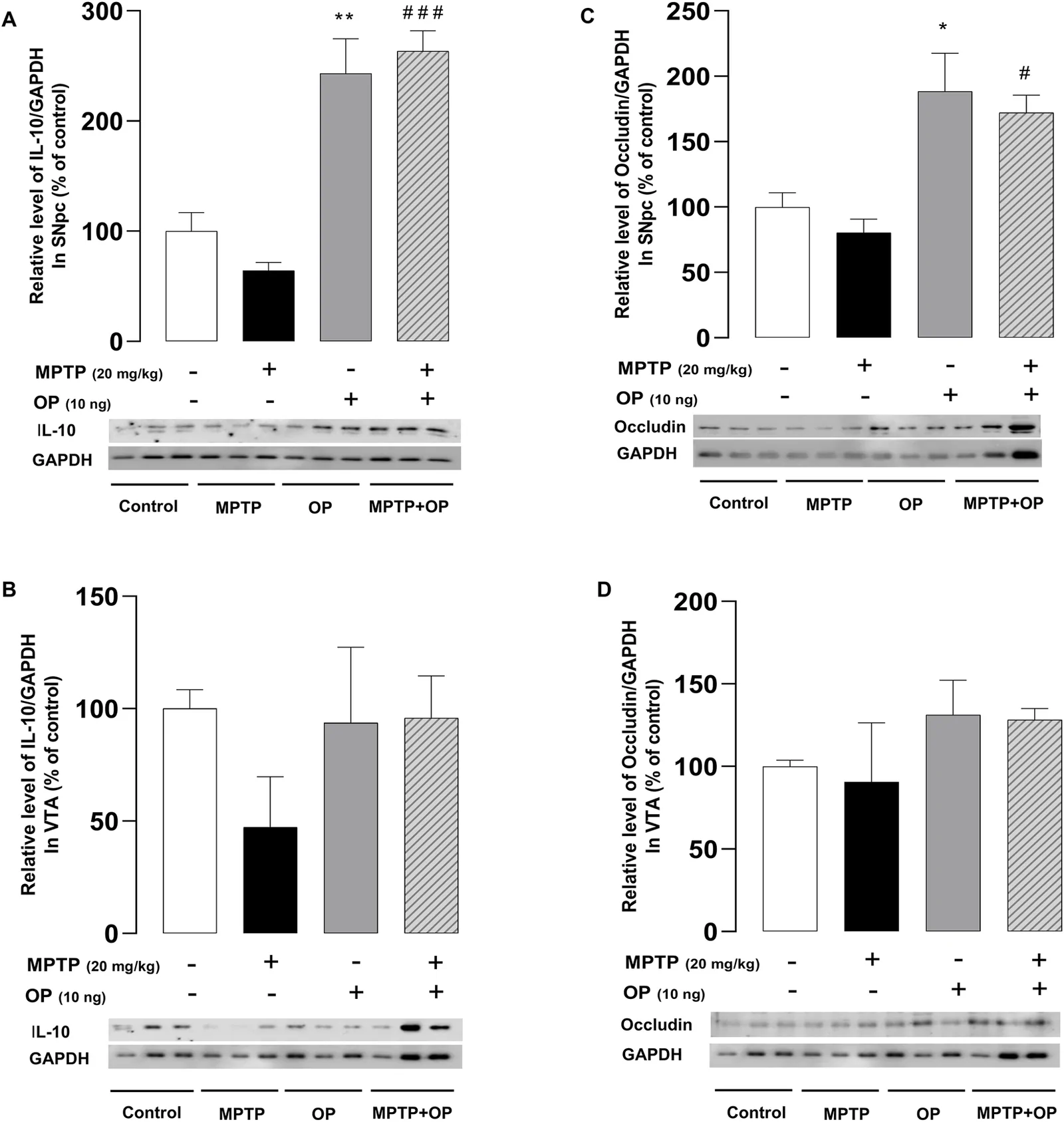
OP enhances anti-inflammatory and junctional protein levels in MPTP-treated mice. Densitometric analysis of IL-10, and occludin protein levels in the SNpc **(A,C)** and VTA **(B,D)** of control, MPTP, OP, and MPTP + OP-treated mice. Representative immunoblots and corresponding quantifications normalized to GAPDH are shown. Data are expressed as mean ± SEM from five animals. Statistical analysis was performed by one-way ANOVA followed by Bonferroni’s *post hoc* test. *p < 0.05, **p < 0.01 vs. saline-treated mice; #*p* < 0.05, ###*p* < 0.001 vs. MPTP-treated mice.

To further investigate the effects of OP on BBB integrity, junction-associated gene expression was analyzed by RT-qPCR in SNpc homogenates. 7 days after MPTP administration, *ZO-1* mRNA levels were reduced (0.79-fold), whereas *claudin-1* and *E-cadherin* expression remained unchanged (Figures 7A–C). OP treatment alone significantly increased *ZO-1* (1.34-fold), *claudin-1* (1.84-fold), and *E-cadherin* (1.5-fold) gene expression compared with control animals. In MPTP-treated mice, OP co-administration maintained elevated mRNA expression of *ZO-1* (1.45-fold vs. MPTP), *claudin-1* (1.99-fold, vs. MPTP) and *E-cadherin* (1.44-fold vs. MPTP) (Figures 7A–C).

**FIGURE 7:**
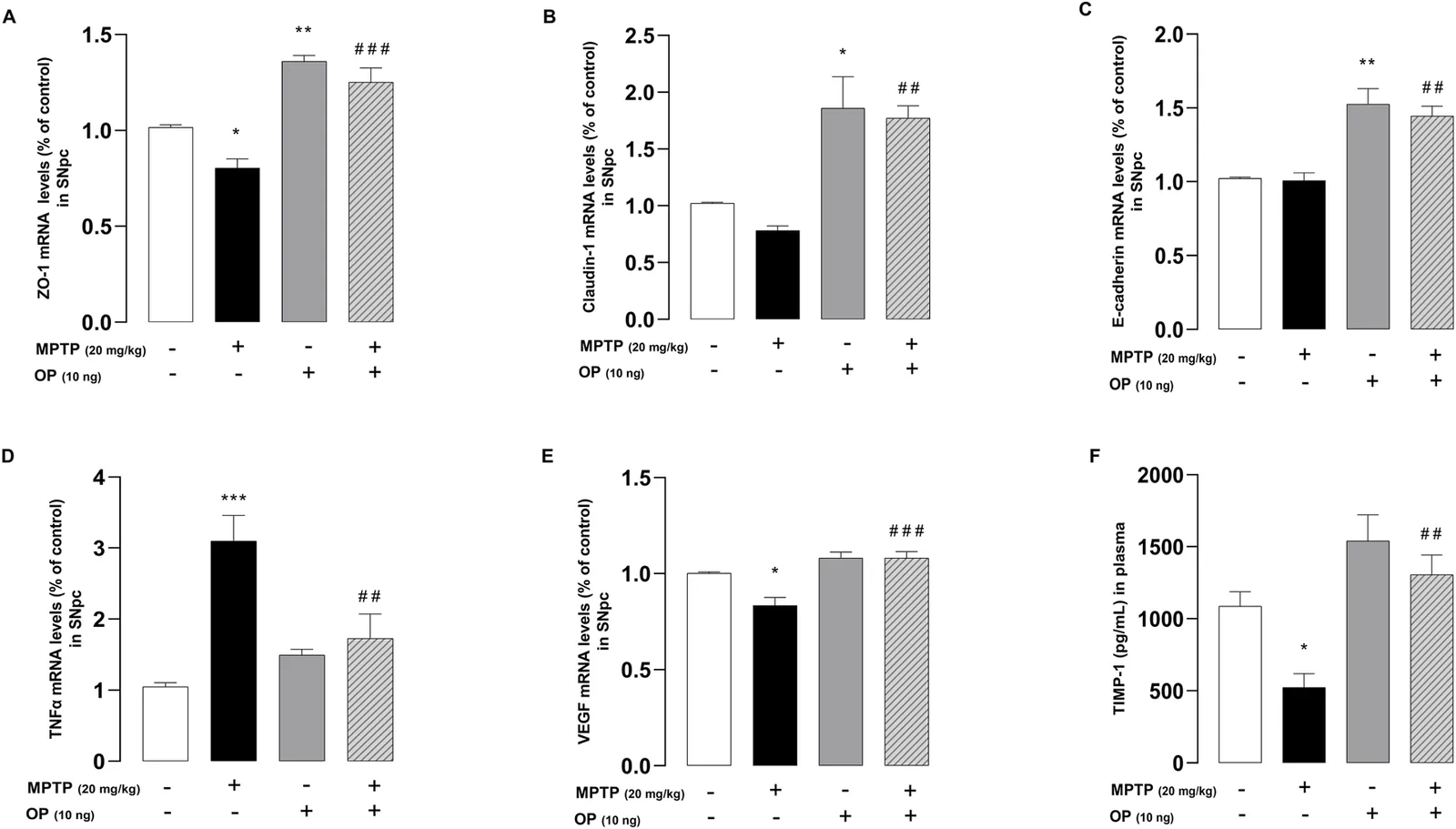
OP regulates junctional and inflammatory gene expression and preserves ECM homeostasis in MPTP-treated mice. mRNA expression levels of **(A)** ZO-1, **(B)** claudin-1, **(C)** E-cadherin, **(D)** TNFα, and **(E)** VEGF in the SNpc of control, MPTP (3 mg/kg × 20 mg/kg), OP (10 ng, 10 μL), and MPTP + OP-treated mice, normalized to GAPDH expression used as an internal control. **(F)** Plasma levels of TIMP-1. Data are expressed as mean ± SEM from five animals for Rt-qPCR and four animals for Elisa. Statistical analysis was performed by one-way ANOVA followed by Bonferroni’s *post hoc* test. *p < 0.05, **p < 0.01, ***p < 0.01 vs. saline-treated mice; ##*p* < 0.01, ###*p* < 0.001 vs. MPTP-treated mice.

Consistent with these effects, tumor necrosis factor-α (TNF-α) mRNA levels were markedly increased in the SNpc of MPTP-treated mice (3.01-fold vs. control; Figure 7D), while vascular endothelial growth factor (VEGF) expression was reduced (0.83-fold vs. control; Figure 7E). OP co-treatment significantly attenuated the MPTP-induced upregulation of *TNF-α* (0.56-fold relative to MPTP) and restored *VEGF* expression (1.25-fold vs. MPTP; Figures 7D,E).

Finally, plasma levels of tissue inhibitor of metalloproteinase-1 (TIMP-1) was measured. MPTP treatment induced a significant reduction in TIMP-1 levels (0.44-fold vs. control), whereas OP co-treatment restored them (1.78-fold vs. MPTP), suggesting improved extracellular matrix homeostasis and vascular integrity.

### OP regulates miRNA expression

In a previous *in vitro* study, we identified several miRNAs strongly associated with PD through bioinformatics analysis (Bourzam et al., 2024). To validate these findings *in vivo*, we analyzed the expression of miR-34b-5p, miR-29a-5p, and miR-21-3p in the SNpc of the different mouse groups. In SNpc of MPTP-treated mice, miR-34b-5p levels remained unchanged compared to controls (Figure 8A), whereas miR-29a-5p and miR-21-3p were significantly upregulated (1.55-fold and 2.24-fold, respectively; Figures 8B,C). Single i.c.v. administration of OP markedly increased miR-34b-5p expression (3.11-fold vs. MPTP) and restored miR-29a-5p and miR-21-3p levels to near-control expression in MPTP-treated mice after 7 days (Figures 8B,C).

**FIGURE 8:**
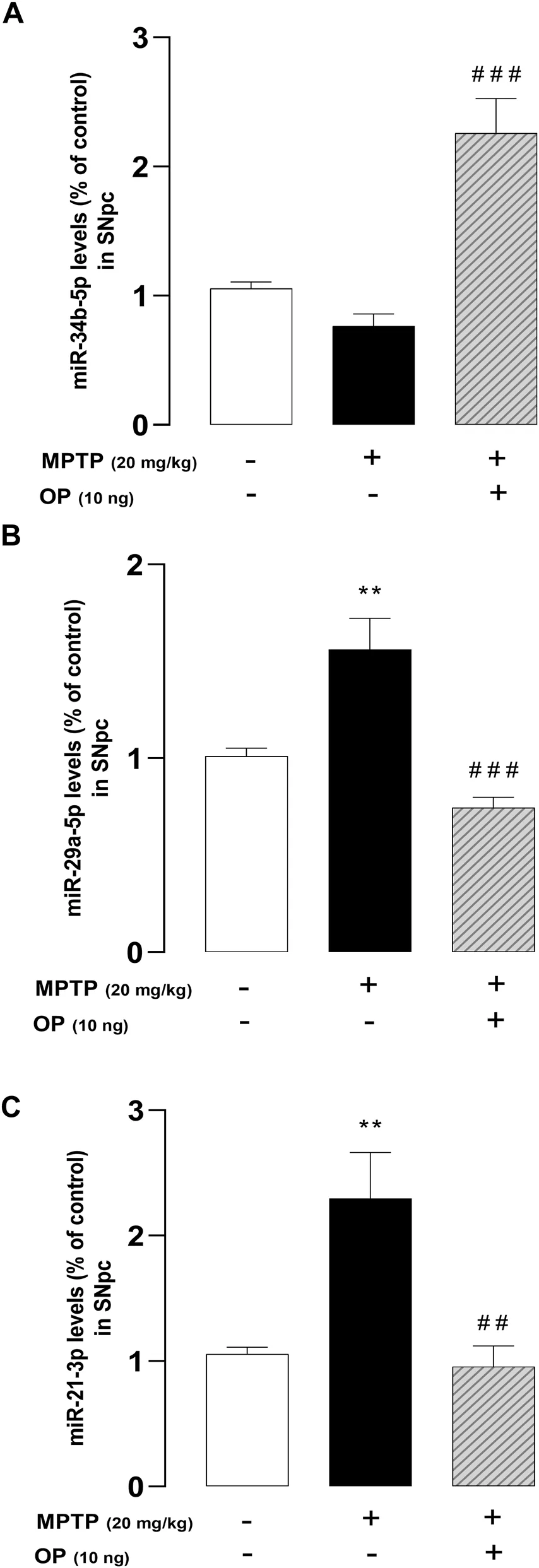
OP modulates miRNA expression in SNpc of MPTP-treated mice. Expression levels **(A)** miR-34b-5p, **(B)** miR-29a-5p, and **(C)** miR-21-3p in the SNpc of control, MPTP (3 mg/kg × 20 mg/kg), OP (10 ng, 10 μL), and MPTP + OP-treated mice, normalized to U6 expression used as an internal control. Data are expressed as mean ± SEM from five animals. Statistical analysis was performed by one-way ANOVA followed by Bonferroni’s *post hoc* test. **p < 0.01 vs. saline treated mice; ##*p* < 0.01, ###*p* < 0.001 vs. MPTP-treated mice.

## Discussion

Previous studies have shown that ODN exerts neuroprotective effects against dopaminergic neurodegeneration in cellular and animal models of PD by modulating pathways associated with oxidative stress and neuroinflammation (Bourzam et al., 2024; Bahdoudi et al., 2018). In the present study, we demonstrate that the ODN analogue OP (Leprince et al., 1998) confers significant neuroprotection in an *in vivo* Parkinsonian mouse model, supporting its potential as a therapeutic strategy to delay dopaminergic neuron degeneration. Consistent with the neurotoxic effects of MPTP, we observed a marked decrease in TH immunoreactivity in both the SNpc and VTA, accompanied by increased markers of neuroinflammation and oxidative stress, as well as significant body weight loss (Bahdoudi et al., 2018; Ma and Rong, 2022). Single administration of OP following MPTP exposure significantly mitigated these alterations, restoring TH protein levels and fluorescence intensity in both the SNpc and VTA, and preventing MPTP-induced body weight loss. Notably, OP, like the authentic peptide ODN (Bahdoudi et al., 2018) exerted robust neuroprotective effects at low doses, preserving dopaminergic neurons from the cytotoxic action of MPTP. Consistently, Atsttrin, a bioengineered protein derived from progranulin, was shown to attenuate MPTP-induced neuroinflammation and partially restore monoamine levels, supporting its potential therapeutic effect in neurodegenerative disorders associated with neuroinflammation process (Poniatowski et al., 2024). Similar properties have been reported for several astroglia-derived neuroprotective peptides, including ADNF, ADNP and its derivative peptide NAP, as well as clusterin, humanin, and dynorphin fragments (Imhof et al., 2006; Lee et al., 2013; Arya et al., 2015; Chen et al., 2021; Galushkin and Gozes, 2025). These peptides exhibit potent neuroprotective potency at femtomolar to picomolar concentrations in PD models and other neurodegenerative conditions, including ischemia and Alzheimer’s disease (Miguel-Hidalgo, 2023). Together, these findings support the classification of ODN and its analogue OP within the emerging class of femtomolar-acting gliogenic peptides with strong neuroprotective potential. The *in vivo* neuroprotective effects of OP on nigrostriatal neurons likely involve both direct actions on dopaminergic neurons and indirect astrocyte-mediated mechanisms, potentially including the release of neurotrophic and protective factors from activated astrocytes.

We next investigated whether the neuroprotective effects of OP involve modulation of oxidative stress. Exposure to MPTP induced a marked increase in ROS production and lipid peroxidation, as evidenced by elevated MDA levels in the SNpc, concomitant with impairment of endogenous antioxidant defenses. In contrast, co-administration of OP significantly attenuated ROS accumulation and MDA formation while restoring catalase activity, indicating a recovery of intrinsic antioxidant capacity. These findings are consistent with previous reports highlighting the antioxidant properties of ODN in both *in vivo* and *in vitro* models of neurodegeneration (Tonon et al., 2020; Masmoudi-Kouki et al., 2020). Notably, administration of ODN during the subacute phase following ischemic stroke has been shown to enhance motor and functional recovery. In Alzheimer’s disease models, amyloid-β accumulation induces oxidative stress, mitochondrial dysfunction, while ODN has been reported to exhibit antioxidant properties (He et al., 2023). whereas ODN deficiency is associated with an increased sensitivity to oxidative stress, exacerbated infarct volume and neuronal cell death (Lamtahri et al., 2021). Furthermore, DBI knockout mice exhibit heightened vulnerability to MPTP-induced neurotoxicity compared with wild-type animals (Bahdoudi et al., 2018). Collectively, the observation that reduced ODN production exacerbates oxidative damage and increases sensitivity to neurodegeneration support the involvement of endogenous antioxidant system in the neuroprotective action of OP against MPTP-induced dopaminergic cell death. In line with this interpretation, inhibition of the endogenous antioxidant system in astrocytes abolish the pro-survival effects of ODN-related peptides under oxidative stress conditions (Ghouili et al., 2018).

The intracellular signaling pathways underlying the neuroprotective effects of OP against MPTP intoxication *in vivo* remains to be fully elucidated. Nevertheless, accumulating evidence indicates that ODN can regulate the expression of Bcl-2 family members to prevent apoptotic cell death evoked by oxidative insults. In agreement with these data, our results demonstrate that OP prevented the MPTP-induced reduction of the anti-apoptotic protein Bcl-2 level and restores phosphorylation of ERK1/2. This effect likely contributes to mitochondrial membrane stabilization, preservation of mitochondrial membrane potential and inhibition of the intrinsic apoptotic pathway (Hamdi et al., 2012a). These findings are in agreement with *in vitro* studies showing that ODN protects glial cells and cerebellar granule neurons from oxidative stress via activation of its metabotropic receptor, engaging PKC/PKA/MAPK pathway (Masmoudi-Kouki et al., 2020). This signaling cascade promotes upregulation of Bcl-2 mRNA expression while inhibiting the pro-apoptotic gene Bax, thereby preventing mitochondrial membrane depolarization and stimulating caspase-3 activity (Kaddour et al., 2013; Hamdi et al., 2012a). Pharmacological evidence further indicates that OP activates ODN metabotropic receptor (Leprince et al., 1998; Leprince et al., 2001; Do Rego et al., 2007), suggesting that its anti-apoptotic effects on dopaminergic neuron are mediated, at least in part, through this receptor and subsequent activation of the MAPK signaling cascade (Hamdi et al., 2012b). Notably, similar mechanisms have been documented for other neuroprotective peptides, such as ADNF, NAP, and dynorphin fragments, which activate ERK and PI3K/Akt pathways, enhance Bcl-2 signaling, and inhibit the mitochondrial intrinsic apoptotic pathway by suppressing Bax expression and caspase-3 activity (Yao et al., 2020).

Beyond its effects on neuronal survival pathways, OP also exerted significant regulatory actions on neuroinflammatory processes. Consistent with our previous *in vitro* findings, ODN counteracts 6-OHDA-induced reduction of IL-10, a key mediator critical of immune homeostasis (Bourzam et al., 2024). *In vivo*, ODN has likewise been shown to attenuate astroglial reactivity and suppress pro-inflammatory gene expression in the SNpc of MPTP-treated animals. Conversely, genetic deletion of its precursor exacerbates MPTP-induced neuroinflammation, leading to increased GFAP-positive astrocytes and elevated expression of IL-6 and TNF-α (Bahdoudi et al., 2018). In agreement with these observations, our results show that MPTP intoxication induced upregulation of TNF-α gene expression alongside inhibition of anti-inflammatory IL-10 gene expression. In contrast, OP administration effectively abolished TNF-α transcript induction and restored IL-10 expression, indicating a shift toward an anti-inflammatory profile. In addition to its anti-inflammatory effects, OP reinforced structural components of the nigrostriatal microenvironment by enhancing the expression of key junctional proteins, including occludin, ZO-1, claudin-1, and E-cadherin within the SNpc. Preservation of these tight junction proteins is increasingly recognized as an important indirect mechanism of neuroprotection, as intact barrier integrity limits the infiltration of peripheral immune factors and circulating toxins that could otherwise amplify local neuroinflammation (Knox et al., 2022; Wu et al., 2023). By stabilizing barrier function, OP may therefore interrupt the pathogenic feed-forward cycle linking BBB disruption to central inflammation and progressive dopaminergic degeneration. Our previous *in vitro* studies further demonstrate that ODN-treated astrocytes exposed to 6-OHDA release elevated levels of anti-inflammatory and trophic mediators, including IL-10, IL-4, MCP-1, L-selectin, CNTF, TIMP-1, and CINC3, collectively promoting a shift toward an M2-like reparative phenotype (Bourzam et al., 2024). Furthermore, we also observed that TIMP-1, a critical regulator of extracellular matrix (ECM) remodeling, was markedly reduced following MPTP exposure but restored to near-baseline levels by OP co-treatment. This finding suggests that OP not only dampens neuroinflammation but also preserves ECM homeostasis, thereby supporting structural repair and neuronal survival. Taken together, these results support the notion that OP acts as a multi-faceted neuroprotective agent, integrating anti-inflammatory reprogramming with reinforcement of junctional integrity and maintenance of ECM stability.

A key experimental aspect of our study lies in the regulation of PD-associated miRNAs by OP. ODN has previously been shown to reverse 6-OHDA-induced dysregulation of apoptosis-related miRNAs (Bourzam et al., 2024). Extending these observations *in vivo*, we demonstrate that MPTP upregulated miR-29a-5p and miR-21-3p while leaving miR-34b-5p unchanged. In contrast, OP normalized miR-29a-5p and miR-21-3p levels and markedly increased miR-34b-5p expression. These results corroborate our earlier bioinformatics analyses, which identify these miRNAs as strongly associated with PD pathogenesis (Bourzam et al., 2024). By modulating their expression *in vivo*, OP appears to promote epigenetic stability, complementing its anti-inflammatory, antioxidant, and anti-apoptotic properties. This regulatory profile aligns with an expanding body of literature positioning specific circulating and tissue miRNAs as both mediators and biomarkers of neurodegeneration (Shaheen et al., 2024; Guévremont et al., 2023; Li et al., 2024). Mechanistically, miR-34b-5p targets genes central to oxidative stress responses, including heme oxygenase-1 (HO-1) and NRF2, thereby contributing to the regulation of ROS homeostasis (Huang et al., 2019). MiR-29a-5p has been implicated in neurite outgrowth during brain repair; however, its overexpression may increase endothelial permeability, compromise BBB integrity, and modulate inflammation through NLRP3 suppression (Zhang et al., 2021). In contrast, miR-21-3p exerts predominantly deleterious effects in PD models: its upregulation enhances TNF-α and IL-6 expression, promotes apoptosis by targeting Bcl-2, and reduces VEGF release (Sims et al., 2017). Consistently, inhibition of miR-21-3p has been shown to decrease cytokine and chemokine release, restore VEGF levels, and increase IL-10 production, thereby exerting anti-inflammatory and neuroprotective actions. In line with these statements, we previously demonstrated that inhibition of miR-21-3p reduces FAS ligand expression in 6-OHDA-treated astrocytes, further supporting its role as a critical regulator of apoptosis (Bourzam et al., 2024). Collectively, these findings indicate that OP-mediated neuroprotection involves not only stabilization of canonical pathogenic pathways but also reprogramming of miRNA networks governing apoptosis, inflammation, and oxidative stress. Notably, the stability of these circulating miRNAs in biofluids and tissues underscores the translational potential of OP as a therapeutic candidate, while positioning the miR-21-3p, miR-29a-5p, miR-34b-5p signature as a promising biomarker platform for early PD diagnosis and personalized therapeutic strategies, particularly since PD-related molecular and cellular alterations arise years before the onset of motor symptoms. From a translational perspective, OP emerges as a promising first-in-class peptide therapeutic candidate. Although i.c.v. administration was employed in the present study to establish its central efficacy, this route remains clinically impractical. In contrast, intranasal delivery of OP represents a more feasible and clinically relevant strategy for future development (Fridhi et al., 2025). This non-invasive approach enables direct nose-to-brain transport via olfactory or trigeminal pathways, effectively bypassing the BBB and allowing therapeutically relevant brain concentrations to be achieved with minimal systemic exposure. Importantly, the pleiotropic properties of OP, particularly its ability to enhance the expression of junctional protein and attenuate neuroinflammatory signaling, suggest that intranasal administration may confer additional benefits by reinforcing BBB integrity while ensuring targeted delivery to vulnerable nigrostriatal circuits. Such dual action could be especially advantageous in the context of neurodegenerative disorders characterized by both barrier dysfunction and chronic inflammation. Future investigations should therefore prioritize comprehensive pharmacokinetic and pharmacodynamic profiling of intranasally administered OP, including detailed assessments of brain bioavailability, regional distribution, and dose-response relationships in chronic models of PD. Moreover, evaluating OP in combination with established dopaminergic therapies, such as L-DOPA, will be essential to determine potential synergistic effects. Establishing its capacity to exert disease-modifying effects in progressive models will represent a critical step toward successful clinical translation.

In summary, this study provides the first *in vivo* evidence that OP, an ODN analogue, confers robust neuroprotection against MPTP-induced nigrostriatal degeneration. This protective effect is mediated through the attenuation of oxidative stress, suppression of neuroinflammatory responses, inhibition of apoptotic signaling, and modulation of disease-relevant miRNA networks. Collectively, these findings position OP as a promising multi-target therapeutic candidate and identify the miR-21-3p/miR-29a-5p/miR-34b-5p as a potential biomarker platform for early detection and personalized therapeutic intervention in PD. Future translational studies should focus on optimizing intranasal delivery strategies, validating efficacy in chronic and progressive disease models, and assessing combination approaches with established dopaminergic therapies such as L-DOPA. These steps will be critical to advancing OP toward clinical development as a novel therapeutical agent.

### Study limitations and future directions

Several limitations warrant consideration. First, although the acute MPTP model is widely used for mechanistic investigation, its acute paradigm does not fully recapitulate the chronic, and progressive nature of human PD. Future studies should therefore determine whether the neuroprotective effects of OP are accompanied by functional improvements using complementary behavioral and motor tests. Further investigations are also needed to evaluate the efficacy of OP in chronic MPTP paradigms or in models based on α-synuclein aggregation, which more closely mimic long-term neurodegenerative processes. Second, while i.c.v. administration provided a robust proof-of-concept for OP central action, this route is not clinically applicable. Subsequent investigations should validate more translational delivery strategies, particularly intranasal or systemic administration, to determine pharmacokinetics, brain bioavailability, and therapeutic efficacy under clinically relevant conditions. Third, although our findings highlight a miRNA signature associated with OP-mediated neuroprotection, further work is required to translate these observations into a viable biomarker platform. Specifically, longitudinal studies should evaluate the temporal expression profiles of the identified miRNAs in plasma across different stages of disease progression in animal models. Crucially, these preclinical findings must be replicated in human cohorts to validate the translational relevance of these circulating miRNAs for early diagnosis, prognosis and patient stratification. Finally, sex-specific differences in OP efficacy were not addressed. Given increasing evidence for sexual dimorphism in PD pathogenesis and treatment responses, future studies should include both male and female subjects to better define potential sex-dependent effects and optimize therapeutic strategies.

## Data Availability

The datasets presented in this study can be found in online repositories. The names of the repository/repositories and accession number(s) can be found in the article/supplementary material.
